# Myosinopathies: pathology and mechanisms

**DOI:** 10.1007/s00401-012-1024-2

**Published:** 2012-08-05

**Authors:** Homa Tajsharghi, Anders Oldfors

**Affiliations:** Department of Pathology, Institute of Biomedicine, University of Gothenburg, Sahlgrenska University Hospital, 413 45 Gothenburg, Sweden

**Keywords:** Myopathy, Myosin, Myosin heavy chain, Mutation, Myosin storage myopathy, Laing distal myopathy, Protein aggregate

## Abstract

The myosin heavy chain (MyHC) is the molecular motor of muscle and forms the backbone of the sarcomere thick filaments. Different MyHC isoforms are of importance for the physiological properties of different muscle fiber types. Hereditary myosin myopathies have emerged as an important group of diseases with variable clinical and morphological expression depending on the mutated isoform and type and location of the mutation. Dominant mutations in developmental MyHC isoform genes (*MYH3* and *MYH8*) are associated with distal arthrogryposis syndromes. Dominant or recessive mutations affecting the type IIa MyHC (*MYH2*) are associated with early-onset myopathies with variable muscle weakness and ophthalmoplegia as a consistent finding. Myopathies with scapuloperoneal, distal or limb-girdle muscle weakness including entities, such as myosin storage myopathy and Laing distal myopathy are the result of usually dominant mutations in the gene for slow/β cardiac MyHC (*MYH7*). Protein aggregation is part of the features in some of these myopathies. In myosin storage myopathy protein aggregates are formed by accumulation of myosin beneath the sarcolemma and between myofibrils. In vitro studies on the effects of different mutations associated with myosin storage myopathy and Laing distal myopathy indicate altered biochemical and biophysical properties of the light meromyosin, which is essential for thick filament assembly. Protein aggregates in the form of tubulofilamentous inclusions in association with vacuolated muscle fibers are present at late stage of dominant myosin IIa myopathy and sometimes in Laing distal myopathy. These protein aggregates exhibit features indicating defective degradation of misfolded proteins. In addition to protein aggregation and muscle fiber degeneration some of the myosin mutations cause functional impairment of the molecular motor adding to the pathogenesis of myosinopathies.

## Introduction

**Fig. 1:**
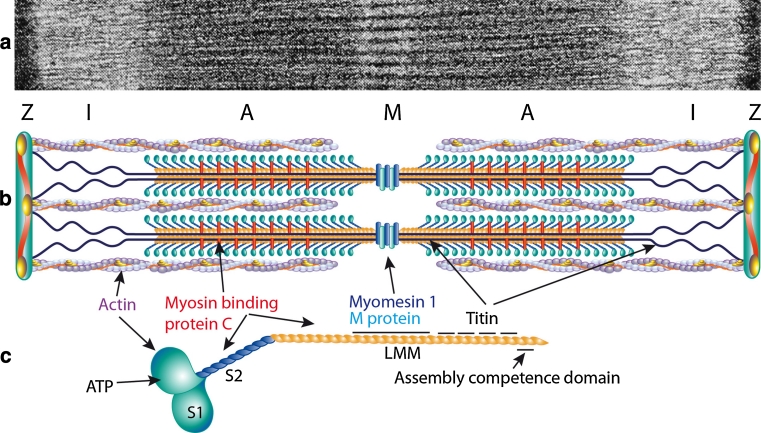
**a** Electron micrograph of a skeletal muscle sarcomere, demonstrating thick and thin filaments and the banding pattern. **b** Schematic drawing of the sarcomere demonstrating the thin filaments composed mainly of actin, tropomyosin and the troponin complex and the thick filaments composed mainly of myosin with the myosin heavy chain (MyHC) globular heads interacting with the thin filaments. **c** Illustration of the MyHC dimer with approximate binding sites for ATP, actin, myosin-binding protein C, myomesin-1, M-protein and titin. The assembly competence domain in the distal rod region is indicated. The different regions of the MyHC (S1, S2 and light meromyosin, LMM) are indicated by *different colors*

Myosin is a highly conserved, ubiquitous protein found in all eukaryotic cells [57]. It acts as a molecular motor that converts chemical energy of ATP hydrolysis into mechanical force for diverse cellular movements such as cytokinesis, phagocytosis, and muscle contraction [58]. Myosins constitute a diverse superfamily and are grouped into different classes including the conventional, or class II, two-headed myosins that form filaments in striated muscle, smooth muscle and non-muscle cells [64]. The class II conventional muscle myosin exists as a hexameric protein composed of two myosin heavy chain (MyHC) subunits and two pairs of non-identical light chain subunits [57, 64]. MyHCs associate into dimers through a coiled-coil interaction along its long tail, which is termed the rod domain (Fig. 1). Dimerization of two heavy chains results in a polar structure with two distinct regions, which provide the motor and filament-forming functions. The amino terminus forms a globular head domain that binds to actin and ATP, which is required for motor activity [56]. The elongated α-helical coiled-coil C-terminal rod domain exhibits filament-forming properties that assemble into thick filaments of the sarcomeres (Fig. 1) [57].

Mutations in MyHC genes have been demonstrated to be an important cause of various myopathies, some of which are associated with protein aggregates in muscle fibers [49, 50].

## Myosin heavy chain isoforms

There are several striated muscle MyHC isoforms encoded by different genes and expressed in a tissue and developmental specific manner [61–63, 84, 85]. In adult human limb skeletal muscle there are three major MyHC isoforms: MyHC I, also called slow/ß-cardiac MyHC, is encoded by *MYH7* and is expressed in slow, type 1 muscle fibers as well as in the ventricles of the heart; MyHC IIa (*MYH2*) is expressed in fast, type 2A muscle fibers and MyHC IIx (*MYH1*) is expressed in fast, type 2B muscle fibers [66] (Table 1). The three different muscle fiber types differ in their contractile and physiological properties, which are partly determined by the different MyHCs. In addition, embryonic and perinatal MyHCs, encoded by *MYH3* and *MYH8*, are expressed during fetal development and also during muscle regeneration [24, 31].

**Table 1:** Myosin heavy chain isoforms expressed in human muscle

Protein	Gene	Muscle fiber type
MyHC IIx/d	*MYH1*	Type 2B
MyHC IIa	*MYH2*	Type 2A Extraocular muscle
Embryonic MyHC	*MYH3*	Fetal development Muscle regeneration
α-cardiac MyHC	*MYH6*	Heart atria
MyHC I β-Cardiac MyHC	*MYH7*	Type 1 Heart ventricles
Fetal MyHC	*MYH8*	Fetal development Muscle regeneration
Smooth muscle MyHC	*MYH11*	Smooth muscle
Extraocular MyHC	*MYH13*	Extraocular muscle

In addition to the common MyHC isoforms expressed in fibers of adult human limb muscles, there are special MyHC isoforms expressed in specific muscles. Muscles of the head and neck such as the masseter muscle show a more diverse expression of MyHC isoforms than limb muscles [52] and very fast contracting fibers found in extraocular muscles express extraocular MyHC isoform (*MYH13*) [54].

The non-muscle conventional class II MyHC genes *MYH9* and *MYH10* encode the non-muscle myosins IIA (MYHIIA) and IIB (MYHIIB), whereas *MYH11* encodes smooth muscle MyHC.

## Myosin heavy chain diseases

The first striated muscle MyHC isoform associated with disease in humans was slow/β cardiac MyHC (*MYH7*) [25]. More than 200 different dominant mutations in *MYH7* have been associated with hypertrophic and dilated cardiomyopathy. Mutations in slow/α-cardiac MyHC (*MYH6*) have been reported to cause hypertrophic and dilated cardiomyopathy and atrial septal defect [14, 16].

*MYH9*-related disorders are autosomal dominant syndromes, variably affecting platelet formation, hearing, and kidney function, and result from mutations in the human nonmuscle myosin IIA heavy chain gene (*MYH9*). They have previously been described as distinct disorders including Sebastian, Fechtner and Epstein syndromes and May-Hegglin anomaly [7, 27].

The first pure skeletal myopathy associated with an identified MyHC mutation was a MyHC IIa myopathy, associated with a dominant *MYH2* mutation that was described in 2000 [40]. Since then, mutations in genes encoding different MyHC isoforms (*MYH2*, *MYH3*, *MYH7* and *MYH8*) have been associated with various skeletal muscle diseases, which are summarized in Table 2.

**Table 2:** Myopathies associated with mutations in skeletal muscle myosin heavy chains

Gene Protein	Disease	Major clinical characteristics	Skeletal muscle pathology
*MYH2* MyHC IIa	Autosomal dominant myopathy with congenital joint contractures, ophthalmoplegia and rimmed vacuoles *OMIM #605637*	Congenital, reversible joint contractures. Ophthalmoplegia. Mild proximal muscle weakness in childhood. Progressive course in some adults affecting ambulation	Rimmed vacuoles with *protein aggregates*, composed of 15-20 nm tubulofilaments, in adults with progressive course and dystrophic muscle changes. Structural alterations with minicores in type 2 fibers in childhood and in mildly affected muscles of adults. Reduced number and small type 2 fibers in some cases.
	Autosomal recessive myopathy with ophthalmoplegia	Mild to moderate muscle weakness, usually mild facial involvement. Ophthalmoplegia	Complete absence of type 2A muscle fibers. Variable, unspecific myopathic changes with fatty infiltration. Type 2B fibers may be lacking.
*MYH3* Embryonic MyHC	DA1 *OMIM #108120* Freeman-Sheldon syndrome, DA2A, *OMIM #193700* Sheldon-Hall syndrome, DA2B, *OMIM #601680*	Multiple congenital joint contractures with predominant distal involvement. No muscle weakness	Minor unspecific changes
*MYH7* MyHC I (ß-cardiac MyHC)	Familial hypertrophic/dilated cardiomyopathy, *OMIM #192600*	Cardiac failure, arrhythmia, sudden cardiac arrest	Irregular structure with cores in type 1 muscle fibers in some patients
	Myosin storage myopathy *OMIM #608358*	Onset from childhood to middle age. Weakness of limb girdle, scapuloperoneal or distal muscles. Mild weakness or severe weakness affecting ambulation	Subsarcolemmal *protein aggregates* in type 1 fibers that reacts with antibodies to myosin but not to desmin. Granular and partly filamentous structure on EM. Myofibrillar disarray.
	Laing early-onset distal myopathy, *OMIM #160500*	Usually onset of distal muscle weakness in childhood, but may be much later. Slowly progressive course with initial weakness of ankle dorsiflexion and “hanging big toe” sign	Fiber size variability, internalized nuclei, frequently small type 1 fibers. Dystrophic changes may occur. Rimmed vacuoles and *protein aggregates* in rare cases, either as inclusions of 15-20 nm tubulo-filaments or as cytoplasmic bodies. Minicores may be frequent.
	Scapuloperoneal and limb girdle syndromes	Scapuloperoneal or limb girdle muscle weakness without morphological features of myosin storage	Unspecific changes including fiber type disproportion
*MYH8* Fetal MyHC	Trismus and pseudocamtodactyly syndrome, DA7 *OMIM#158300*	Congenital contractures of hands, feet and jaws with trismus and hand and foot deformities with pseudocamptodactyly	Not described

*EM* Electron microscopy

Loss of thick filaments and myosin, but preserved thin filaments can be seen as an unspecific alteration in various conditions such as dermatomyositis but more typically in critical illness myopathy [37]. This loss of myosin and other proteins associated with the thick filaments is frequently triggered by systemic corticosteroid hormone treatment, postsynaptic block of neuromuscular transmission and prolonged mechanical ventilation. The loss of myosin can be identified by electron microscopy showing a characteristic disappearance of thick filaments or by measuring the ratio of myosin to actin after separation of muscle proteins by gel electrophoresis.

## Myopathies associated with developmental MyHC isoforms, *MYH3* and *MYH8*

The essential roles of embryonic and fetal MyHC isoforms for normal fetal development has been highlighted by the identification of dominant *MYH3* and *MYH8* mutations associated with distal arthrogryposis (DA) syndromes. In addition to common DA manifestations such as clubfeet and clenched fists these syndromes frequently include also decreased movement of proximal joints, facial dysmorphism and other manifestations [8, 26].

At least 18 mutations in the globular head and rod domains of *MYH3* have been associated with DA1, DA2A (Freeman–Sheldon syndrome) and DA2B (Sheldon–Hall syndrome) [3, 71, 79]. The amino acid at position 672 (R672) is the most frequently mutated residue associated with Freeman–Sheldon syndrome indicating a mutational hotspot of this residue [79]. One mutation has been identified in *MYH8* in several unrelated families with the trismus-pseudocamptodactyly syndrome [78, 82]. Interestingly, this mutation in *MYH8* affects the residue R764 (R764Q), which is paralogous to R672 in *MYH3*. The embryonic and fetal MyHCs are expressed during embryonic and fetal development [24, 31] and it has been suggested that mutations in *MYH3* and *MYH8* cause a developmental myopathy resulting in reduced fetal movement and joint contractures [71, 79]. There are few reports on muscle pathology in distal arthrogryposis associated with MyHC mutations. These reports are limited to muscle biopsies from children and adults, which show minor unspecific changes [71].

## Myopathies associated with MyHC IIa, *MYH2*

Dominant as well as recessive mutations in *MYH2* that cause myopathy have been identified.

Autosomal dominant MyHC IIa myopathy, which has also been referred to as “autosomal dominant myopathy with congenital joint contractures, ophthalmoplegia and rimmed vacuoles”, was originally identified as a muscle disorder in western Sweden [19]. The disease was mapped to chromosome 17p13.1 [39] and later demonstrated to be caused by a heterozygous missense mutation in *MYH2* encoding MyHC IIa [40]. The mutation changes the highly conserved and negatively charged glutamate at position 706 to the positively charged lysine (E706K). The mutated residue is located in the SH1 helix in the core of motor domain, which is highly conserved through evolution.

A prenatal onset of the disease was indicated by multiple joint contractures that were present at birth in the majority of the patients. The joint contractures preferentially involved the fingers and/or hips and generally resolved early in childhood. Hypotonia was not a prominent feature and the early development was normal. External ophthalmoplegia was present in all patients, ranging from a slight impairment of upward gaze in affected children to a generalized ophthalmoparesis in some adult cases. The muscle weakness and atrophy predominantly involved proximal muscles of the shoulder and pelvic girdles, and also back and hand muscles. Atrophy of the quadriceps muscles was prominent in the more severely affected adults. The muscle weakness was remarkably variable. Most children and adolescents and some adults were mildly affected, whereas some adults had experienced progressive muscle weakness affecting ambulation from age 30 to 50 years. While mildly affected cases had normal serum creatine kinase (s-CK) levels, it was slightly elevated in the family members showing a progressive course. A fine action hand tremor has been noticed in several cases.

**Fig. 2:**
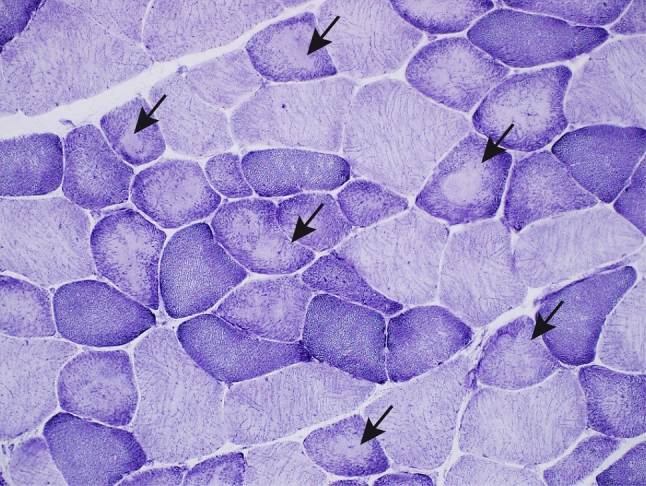
Dominant myosin IIa myopathy. Biopsy of the deltoid muscle of a 38-year-old man showing alterations of the type 2A fibers (*arrows*). NADH-tetrazolium reductase

**Fig. 3:**
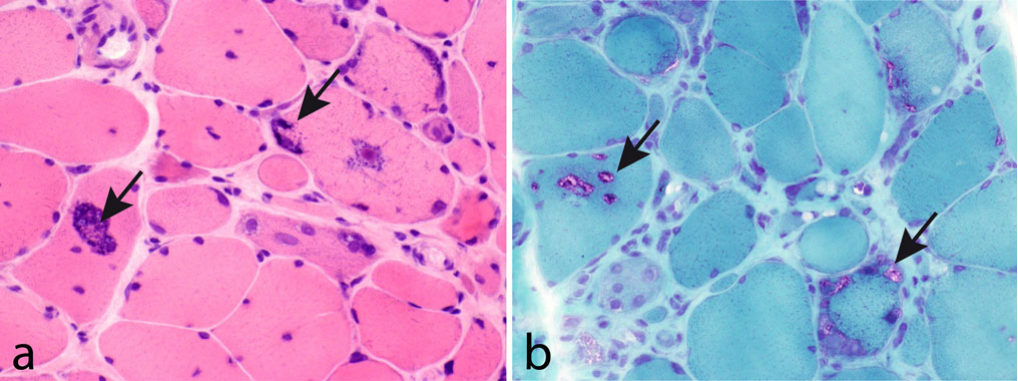
Dominant myosin IIa myopathy. Biopsy of the quadriceps muscle of a 38-year-old man demonstrating variability of fiber size, increased interstitial connective tissue, and frequent fibers with rimmed vacuoles. **a** Hematoxylin and eosin; **b** Gomori trichrome

**Fig. 4:**
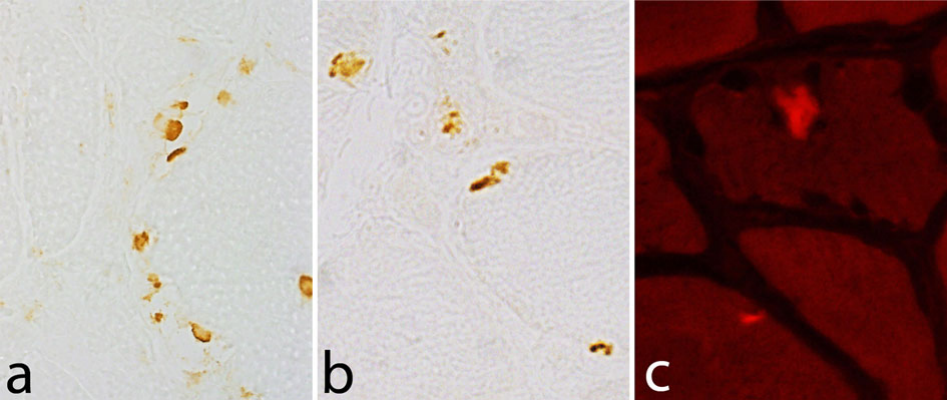
Dominant myosin IIa myopathy. Several muscle fibers show inclusions that are immunoreactive to antibodies against p62 (**a**) and ubiquitin (**b**). Amyloid is present in a few inclusions as revealed by Congo staining and fluorescence microscopy with Texas red filter (**c**)

**Fig. 5:**
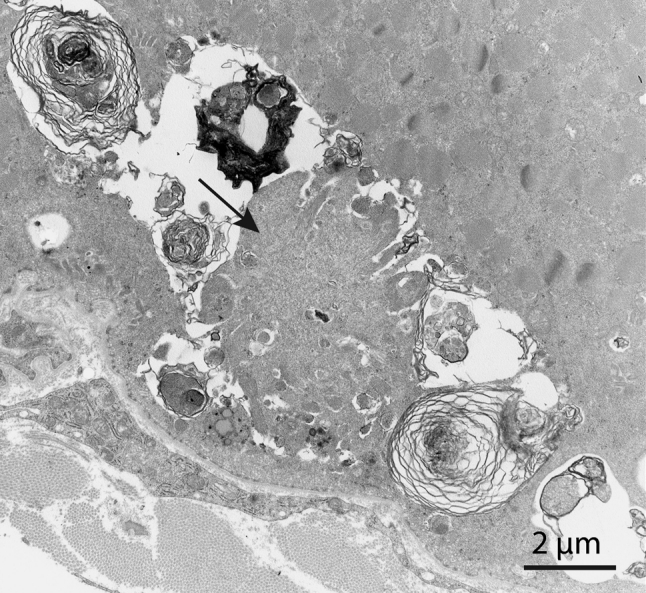
Dominant myosin IIa myopathy. Electron microscopy reveals a filamentous inclusion (*arrow*) associated with a rimmed vacuole with degradation products

**Fig. 6:**
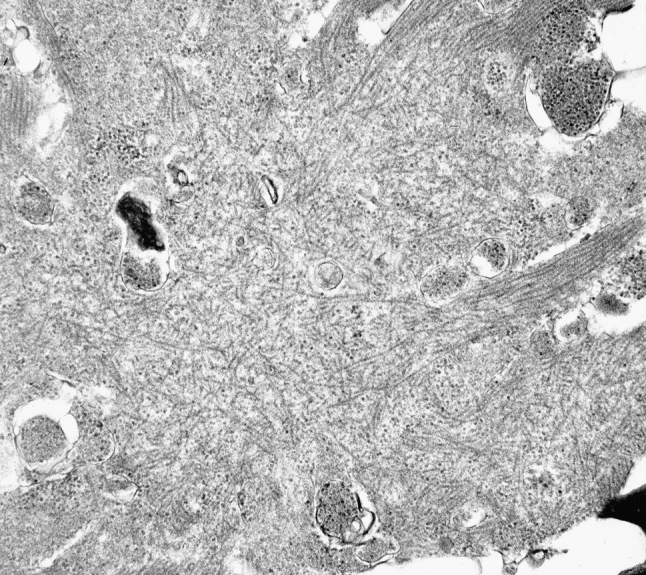
Dominant myosin IIa myopathy. Electron microscopy demonstrating an inclusion composed of 15–20 nm tubulofilaments

**Fig. 7:**
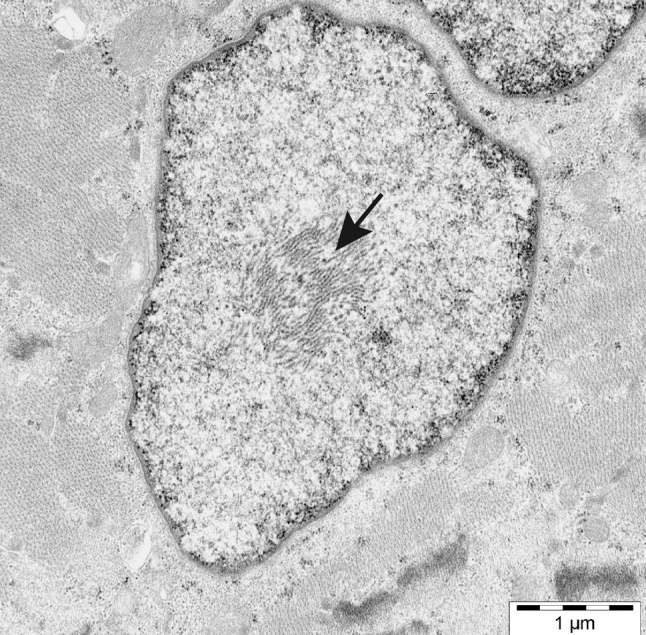
Electron micrograph illustrating intranuclear filaments (*arrow*) in dominant myosin IIa myopathy

Muscle pathology was highly variable, also in different muscles of the same individual. A consistent finding was a predominant involvement of type 2A muscle fibers (Fig. 2). In children and mildly affected adults the type 2A fibers were reduced in number, frequently smaller than normal and often demonstrating disorganization of myofibrils, similar to what is observed in multi-mini core disease [19]. Adults with progressive course and increased s-CK levels demonstrated dystrophic muscle pathology with increased interstitial fat and connective tissue. The presence of rimmed vacuoles in many fibers (Fig. 3) in the more advanced cases was the reason at that time to consider this myopathy as a variant of hereditary inclusion body myopathy (hIBM3). The number of rimmed vacuoles was variable also in the severely affected cases. These rimmed vacuoles are associated with inclusions that stain positively for ubiquitin and sequestosome 1 (SQSM1/p62) (Fig. 4a, b). SQSM1/p62 is a component of many disease-associated intracellular multiprotein aggregates and a useful marker for the identification of inclusions in sporadic inclusion body myositis [18, 47]. A few inclusions show congophilia (Fig. 4c). Inclusions of tubulofilaments measuring 15–21 nm in diameter are present, usually in association with rimmed vacuoles (Figs. 5, 6). Intranuclear filaments can also be observed (Fig. 7).

The severity of the disease was apparently related to the amount of expressed MyHC IIa protein in muscle [75]. Severely affected individuals and muscles showed a large proportion of type 2A muscle fibers, and the fibers with rimmed vacuoles expressed MyHC IIa. Studies on the effects of endurance training have been performed [69, 74]. An 8-week endurance training program on a stationary exercise bicycle had no adverse effects. The peak watt and walking speed increased, but there was no significant increase in muscle strength or isometric endurance [69]. The training program resulted in a shift in expression from fast to slow MyHC isoforms. There was no significant change in the MyHC IIa expression, but frequent hybrid fibers expressing more than one MyHC isoform were identified [74].

Patients with recessive MyHC IIa mutations have recently been identified. The so far published cases are homozygous or compound heterozygous for truncating mutations in *MYH2*, accompanied by complete loss of MyHC IIa protein and absence of type 2A muscle fibers [70]. The clinical picture was surprisingly mild with minor or moderate generalized muscle weakness including facial muscle weakness. A consistent finding was external ophthalmoplegia, which was only occasionally associated with ptosis. Magnetic resonance imaging (MRI) of lower limb muscles in two cases demonstrated selective involvement of certain muscles with fatty infiltration. There was predominant involvement of medial gastrocnemius in the lower legs, combined with predominant involvement of the semitendinosus, gracilis and vastus lateralis muscles in the thigh.

Muscle biopsy demonstrated in addition to complete loss of MyHC IIa protein, unspecific myopathic changes with fiber size variability, internalized nuclei and interstitial fatty infiltration [70]. Some muscle biopsy samples demonstrated type 1 fiber uniformity. Unlike the dominant MyHC IIa myopathy, no rimmed vacuoles or protein aggregates have been identified.

## Myopathies associated with slow/beta MyHC, *MYH7*

Overall, mutations in *MYH7* are predominantly missense mutations located in the globular myosin head, potentially affecting the binding sites for actin or nucleotides but there are also numerous mutations in the rod region. More than 200 different mutations have been associated with either hypertrophic or dilated cardiomyopathy, but there are also pure skeletal myopathies and combination of myopathy and cardiomyopathy.

### Myosin storage myopathy

**Fig. 8:**
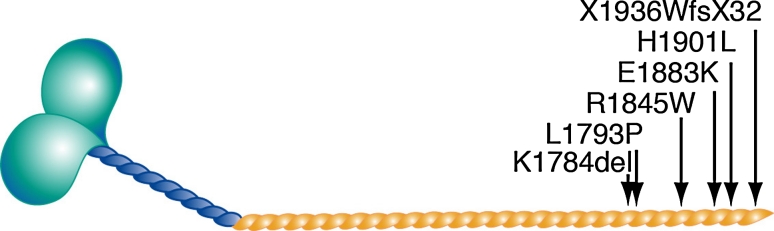
Mutation in *MYH7* associated with myosin storage myopathy. They are all located in the distal rod region of the MyHC

Myosin storage myopathy is a protein aggregate myopathy associated with myosin accumulation [76]. Mutations that cause myosin storage myopathy are located in the distal end of the tail of slow/β cardiac MyHC, corresponding to exons 37–40 of *MYH7* (Fig. 8).

A dominant missense mutation changing the highly conserved and positively charged arginine at position 1845 to the uncharged, aromatic tryptophan (R1845W) was the first mutation identified in myosin storage myopathy. This mutation has been reported in several unrelated cases indicating that the C nucleotide at position 5533 is a mutational hotspot [32, 34, 55, 65, 76]. Two additional dominant missense mutations, H1901L [10] and L1793P [22], have later been reported to cause myosin storage myopathy. Recently, a heterozygous A to G transition in exon 40 of *MYH7* was reported in several individuals of a five-generation family [51]. The mutation is predicted to change the terminal stop codon (TAG) to a tryptophan (W), resulting in an elongation of the C-terminal tail region (X1936WfsX32). Another dominant in frame deletion of the amino acid lysine at position 1784 (K1784del) in exon 37 of *MYH7* has recently been reported in a case with myosin storage myopathy [68]. Myosin storage myopathy combined with cardiomyopathy was reported in three siblings homozygous for a missense mutation (E1883K), but with apparently unaffected parents [72]. Mutations in the distal rod region of slow/β cardiac MyHC associated with myosin storage myopathy may thus be either dominant or recessive.

Myosin storage myopathy was first reported as a familial myopathy with probable lysis of myofibrils in type 1 fibers by Cancilla et al. [13]. The inclusions were described as hyaline bodies because of their unstructured appearance at light microscopy [15], and the term hyaline body myopathy was used in some subsequent case reports. After the discovery that the storage material consists of mainly myosin heavy chain immunoreactive material and that the mutated gene is the slow/β cardiac MyHC gene, *MYH7*, the term myosin storage myopathy was introduced [76] and has been adopted and used in most subsequent reports.

The clinical manifestations are highly variable ranging from no weakness to severe impairment of ambulation [9, 11, 13, 15, 34, 42, 55, 59, 65, 68, 76, 81]. This variability can also be present within a family [11, 55, 81]. Onset is usually in childhood but may be much later. Presenting signs and symptoms are often delayed motor milestones, difficulties in climbing stairs or running and a waddling gate. The distribution of muscle weakness is usually proximal in upper extremities with difficulties in lifting the arms above the shoulder level and scapular winging has been described in many cases. In the lower extremities a distal involvement with foot drop is common and pseudo-hypertrophy of the calves is frequently encountered. Apart from a scapulo-peroneal distribution of weakness, a predominant limb-girdle weakness can be seen in some cases [13, 15]. The course is usually slowly progressive and scoliosis sometimes supervenes [11, 68] and some patients need assisted ventilation.

Evidence of cardiomyopathy associated with the myopathy is usually not found. However, in one woman with muscle weakness since age 30, hypertrophic cardiomyopathy was diagnosed at age 51 [81]. Her 24-year-old daughter presented with cardiac failure already at age 3 months and was diagnosed with signs of left ventricular non-compaction. Three severely affected siblings homozygous for apparently recessive mutations had myosin storage myopathy and hypertrophic cardiomyopathy with cardiac failure that was lethal in two cases [72].

Magnetic resonance imaging (MRI) of muscle in myosin storage myopathy in ten individuals from one family demonstrated in the lower limbs early involvement of the biceps femoris and semimembranosus muscles and relative sparing of the semitendinosus in the posterior compartment of the thigh [55]. In the distal muscles of the legs there was a predominant involvement of the medial gastrocnemius, tibialis anterior, extensor hallucis longus and extensor digitorum longus muscles. In the upper limbs there was a predominant involvement of the deltoid muscle. There was no asymmetry.

**Fig. 9:**
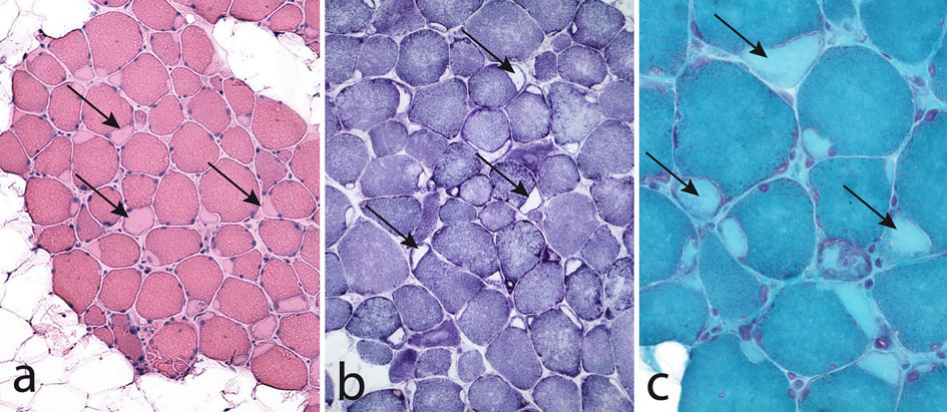
Myosin storage myopathy. There is fatty infiltration and numerous muscle fibers with subsarcolemmal accumulation of material (*arrows*) that stains faintly red by hematoxylin–eosin (**a**) is unstained by NADH-tetrazolium reductase (**b**) and stains *light green* in Gomori trichrome (**c**)

**Fig. 10:**
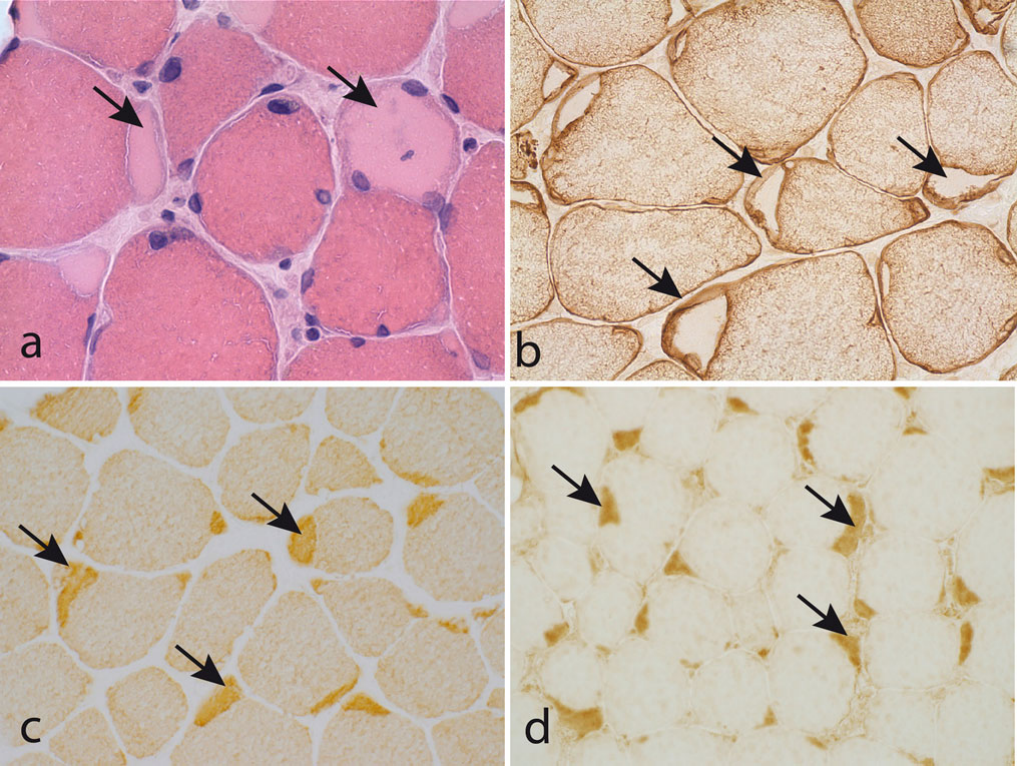
Myosin storage myopathy. The inclusions (*arrows*) appear unstructured in hematoxylin-eosin (**a**) and are surrounded by a rim of desmin (**b**). The inclusions are immunoreactive with antibodies against slow/β cardiac MyHC (**c**) and ubiquitin (**d**)

**Fig. 11:**
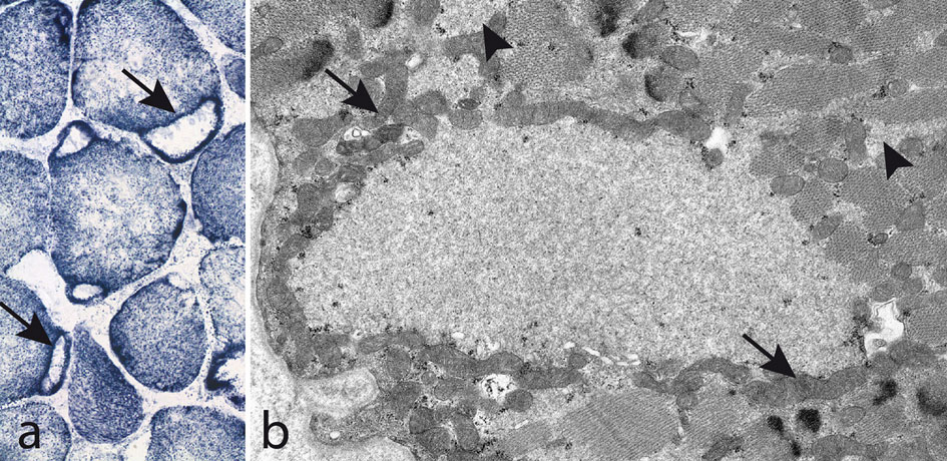
Myosin storage myopathy. The inclusions are surrounded by a rim of increased succinate dehydrogenase activity (*arrows*) (**a**), which corresponds to the presence of numerous mitochondria (*arrows*) around but not within the inclusions as revealed by electron microscopy (**b**). There is also storage material between surrounding myofibrils (*arrow heads*)

**Fig. 12:**
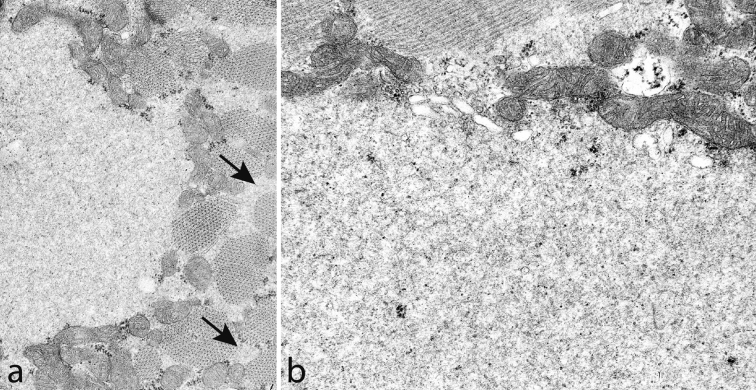
Myosin storage myopathy. Electron microscopy of storage material. **a** The inclusions are not limited by any membrane but instead the storage material can be seen surrounding the adjacent myofibrils (*arrows*). **b** The storage material appears granular and party filamentous and some glycogen particles are also present

Muscle biopsy demonstrates the characteristic subsarcolemmal accumulation of material that is slightly eosinophilic and stains light green in trichrome but there is no NADH-tetrazolium-reductase staining (Fig. 9). By light microscopy the material looks completely unstructured giving it a hyaline appearance (Fig. 10a). There is no immunoreactivity of dystrophin, sarcoglycans or merosin in relation to the stored material, but immunostaining of desmin and also of αβ-crystalline may show a rim of intense staining around the stored material (Fig. 10b) [11, 55]. The stored material is restricted to type 1 fibers and usually demonstrates myofibrillar ATPase activity and immunoreactivity to slow/β cardiac MyHC (Fig. 10c) [11, 51, 55, 68, 76, 81]. However, there are also reports indicating that the accumulated material may be immunoreactive to antibodies against fast myosin isoforms [15, 65]. We have found the stored material to be immunoreactive with antibodies against ubiquitin (Fig. 10d), which is different from a previous report [11], but we have not identified any p62 positive material. The stored material shows no cytochrome *c* oxidase or succinate dehydrogenase activity (Fig. 11a), but there is usually a rim of increased enzyme activity in the periphery corresponding to mitochondria (Fig. 11b). The inclusion bodies are not limited by a membrane but the stored material can be seen between partly disintegrated myofibrils in the vicinity of the main storage body (Figs. 11b, 12a). At higher magnification the storage material has a granular appearance and a few abortive filaments can be seen (Fig. 12b). The inclusions in myosin storage myopathy differ from those in cap disease as the latter contain remnants of sarcomeres with thickened and fragmented Z-lines and thin filaments. The caps show positive immunostaining to desmin whereas the inclusions in myosin storage myopathy are unstained.

### Laing (Gowers–Laing) distal myopathy

**Fig. 13:**
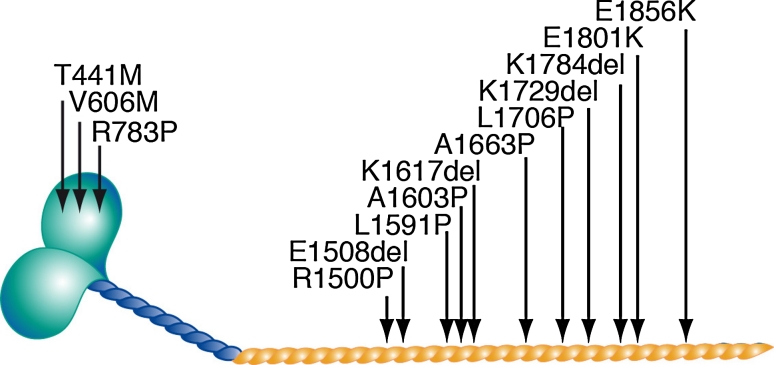
Mutations in *MYH7* associated with distal myopathy. They are mainly located in mid-rod region of the MyHC, but some are located in the globular myosin head and the distal rod

Most patients reported with Laing distal myopathy have dominant mutations in *MYH7* located in exon 32-36 in the mid region of the MyHC rod including: R1500P, E1508del, L1591P, A1603P, K1617del, A1663P, L1706P and K1729del (Fig. 13) [21, 43, 44, 80]. The L1729del mutation has been identified in multiple unrelated patients [44, 45]. However, *MYH7*-associated distal myopathy is not restricted to mutations in this region. Three mutations have been identified in the globular head (T441M, V606M and R783P) [20, 29, 53], and three mutations have been identified in the distal rod region overlapping with the myosin storage myopathy region (E1801K, E1856K and K1784del) (Fig. 13) [77, 80]. Five of these six mutations were associated with distal myopathy and cardiomyopathy. Muscle morphology was not reported in the patients with the three distal mutations (K1784del, E1801K and E1856K) [77, 80] and it cannot be excluded that they were cases of myosin storage myopathy, since the K1784del mutation has been associated with myosin storage myopathy [68]. For classification of diseases, which are associated with mutations in the LMM of slow/β cardiac MyHC and may show clinical overlap, it is essential to perform muscle biopsy.

Laing distal myopathy was first linked to chromosome 14q11 [35, 83], and it was later demonstrated to be caused by mutations in *MYH7* [43]. Most cases have presented early in childhood and hence the term “early-onset” distal myopathy has been used in several reports. Onset is, however, not always in childhood but can occur with a range from congenital to 50 years [35, 36, 41, 44, 77, 83]. The clinical course in typical cases starts with early-onset weakness of ankle dorsiflexors and a “hanging big toe” sign. Calf hypertrophy may be present. During the slowly progressive course finger extensor and neck flexion weakness and later also proximal limb and facial muscle weakness supervenes. Patients usually remain ambulant. Serum CK levels are normal or moderately elevated. Cardiac involvement is usually not a feature of Laing distal myopathy but there are a few exceptions with diseases associated with *MYH7* mutations and presenting with distal myopathy as well as cardiomyopathy [20, 29, 53, 80].

Muscle MRI has demonstrated early changes in the anterior compartments of the lower leg especially the extensor hallucis longus and tibialis anterior muscles [21, 44, 77]. Later in the course and in more severely affected patients additional muscles in the thigh may be involved.

**Fig. 14:**
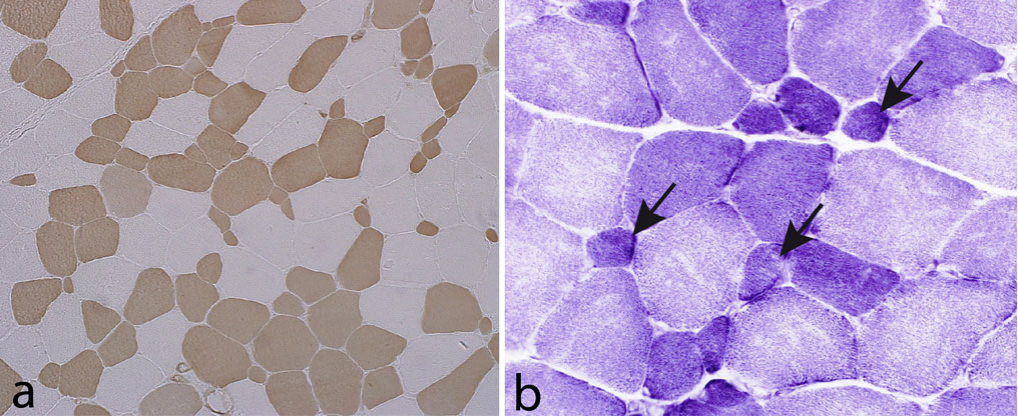
Tibialis anterior muscle of a 7-year-old boy with *MYH7*-associated distal myopathy. Many of the type 1 fibers are small (*arrows*). **a** Myofibrillar ATPase, pH 4.3 and **b** NADH-tetrazolium reductase

Muscle pathology has been reported in most case reports and more systematically in patients from a few families [36, 44, 83]. The pathological changes are, unlike those in myosin storage disease, variable and unspecific. Common findings are predominance of type 1 fibers and numerous small type 1 fibers (Fig. 14), which may appear as fiber type disproportion. Internal nuclei are usually present and structural abnormalities, especially minicores, in addition to mitochondrial abnormalities are common. Muscle fiber necrosis can be seen but is usually not conspicuous. The muscle may show severe atrophy with fat and connective tissue replacement. Protein aggregates are usually not seen, but rimmed vacuoles and filamentous inclusions of the same type as in MyHC IIa myopathy and inclusion body myositis have been observed [41, 77]. In one case cytoplasmic bodies and myofibrillar alterations similar to those seen in myofibrillar myopathies were reported [77].

### Other skeletal muscle phenotypes associated with *MYH7* mutations

Although the most important skeletal muscle manifestations of *MYH7* mutations are referred to as either myosin storage myopathy or Laing distal myopathy several cases cannot be classified into these groups because they either do not display distal muscle weakness or do not exhibit hyaline bodies. Muscle biopsy of some patients with mutations that are known to cause myosin storage myopathy may not reveal the pathognomonic changes, but instead unspecific changes [55] or alterations diagnostic for congenital fiber type disproportion [51]. A few patients with *MYH7* mutations that otherwise cause distal myopathy could be classified as having limb-girdle syndrome or scapuloperoneal myopathy thus overlapping with the clinical features of myosin storage myopathy [44]. In some but not all patients with cardiomyopathy due to *MYH7* mutations, but without overt muscle weakness, cores may be identified in skeletal muscle fibers [23].

## Pathogenesis

### *MYH3* mutations and distal arthrogryposis

A correct understanding of the cause of distal arthrogryposis due to *MYH3* mutations would require examination of muscle pathology during the developmental period when *MYH3* is expressed. Since *MYH3* mutations associated with distal arthtrogryposis are dominant, it may be hypothesized that they affect muscle function during early development through either haploinsufficiency with insufficient dosage of a functional embryonic MyHC or a dominant negative effect of the mutated allele. Most of the *MYH3* mutations are missense, but it is not known whether the mutant allele is expressed. Although the hemizygous loss of MyHC IIa expression is tolerated well in individuals with heterozygous *MYH2* null mutations [70], the situation may be different with embryonic MyHC, since it is a predominant MyHC isoform expressed during an early period of development. Another possibility would be that *MYH3* mutations impose dominant negative effects by functional or structural alterations as seen in dominant mutations affecting the adult MyHC isoforms. However, studies on disease models are needed to pinpoint the pathogenesis of developmental myopathy caused by *MYH3* mutations.

### The MyHC IIa E706K mutation

The E706K mutation associated with the autosomal dominant MyHC IIa myopathy has been studied at the molecular level in different systems. The mutated residue is situated in the SH1 helix in the core of myosin motor domain, which is strongly conserved throughout the myosin class II family. The SH1 helix plays a central role in the conformational changes of the myosin head during the ATP cycle.

Several studies have suggested impaired functional properties as the primary molecular defect of the E706K mutated MyHC protein. In vitro motility studies on myosin IIa isolated from single muscle fibers of patients carrying the E706K mutation clearly showed a marked reduction of speed [38]. Furthermore, studies on the motor function of E683K mutant myosin of *Dictyostelium* *discoideum,* which is equivalent to the human MyHC IIa E706K mutation, demonstrated a threefold reduction of the ATP hydrolysis step followed by the slower acto-myosin dissociation [87]. Consequently, these effects lead to a reduced velocity of contraction. A transgenic *C. elegans* was generated to simulate MyHC IIa myopathy in order to assess the functional or structural effects of the E706K mutation [73]. Worms that were null mutant for *UNC*-*54* (encoding the major *C. elegans* myosin heavy chain expressed in body wall muscle) were partly rescued by transfection with wild type *UNC*-*54*. Transfection with a construct encoding *UNC*-*54* with an E710K mutation corresponding to the human MyHC IIa E706K mutation resulted in restored thick filaments but completely paralyzed worms [73]. In addition, mutations in SH1 helix of nonmuscle class II MYHIIA, encoded by *MYH9* (R702C and R705H), have been associated with a group of inherited giant-platelet disorders in several families [28]. Association of diseases with mutations in SH1 helix of myosin in both muscle and non-muscle MyHC illustrates the crucial role of this thiol region in the head of myosin for proper function of the protein. These different studies all point to a severe defect of the molecular motor function caused by the E706K mutation.

Structural changes in the muscle fibers and muscle fiber loss are likely to be an additional mechanism causing muscle weakness. Morphological studies on muscle biopsy specimens have demonstrated a clear correlation between the expression of the mutated MyHC IIa and dystrophic changes in muscle [75]. The high expression of mutant protein combined with aging may cause defective elimination of misfolded proteins, resulting in accumulation of protein aggregates and secondary muscle fiber degeneration. This hypothesis is supported by the accumulation of ubiquitinated and p62-labeled protein aggregates in vacuolated muscle fibers. This mechanism could be similar to what is seen in inclusion body myositis although the upstream events are different [6, 46, 47]. The same mechanism could also be involved in the muscle degeneration and protein aggregation seen in advanced cases of Laing distal myopathy.

In a recent investigation of the molecular background of the mouse mutant *ariel* a recessive missense mutation, L342Q, in the motor domain of *Myh4* was found to cause a myofibrillar myopathy-like phenotype with protein aggregates in fast MyHC IIb fibers [33]. The mutant protein formed also aggregates in COS7 cells and did not incorporate into thick filaments of C2C12 cells. In *ariel* heterozygotes, which showed no weakness or aggregates, the level of mutant protein was only 7 %, indicating efficient degradation of the defective protein prohibiting protein aggregation. *MYH4* is not expressed in human limb muscle. However, mRNA but not protein has been identified in human masseter muscle [30]. A functional equivalent of mouse MyHC IIb is MyHC IIx (*MYH1*) in humans. So far no disease has been associated with human *MYH1* mutations. Protein aggregation with features similar to those in myofibrillar myopathy and in the *ariel* mouse was identified in one patient with a mutation in the rod region of MyHC I (*MYH7*) mutation [77].

### MyHC IIa null mutations

The selective muscle involvement of certain muscles with fatty infiltration and loss of type 2A muscle fibers with interstitial fatty infiltration in patients with MyHC IIa null myopathy indicate that complete loss of one adult MyHC cannot be compensated for by other MyHC isoforms [70]. This is further supported by studies on strains of mice null for different MyHC isoforms indicating that different isoforms of MyHC are functionally unique and cannot substitute for one another [1, 2, 60]. Mice MyHC IIb or IId null strains are viable but exhibit growth and muscle defects with significant decreases in body mass and mean muscle mass. Although both strains showed evidence of skeletal muscle pathology, the extent and the pattern of affected muscles did not correlate with the abundance and distribution of the two MyHC isoforms in normal mice. Together these observations indicate that MyHC isoforms impose unique structural and functional roles and support the hypothesis that MyHC isoforms are unable to replace one another.

### Mutations in the LMM region of slow/*β*-cardiac MyHC

**Fig. 15:**
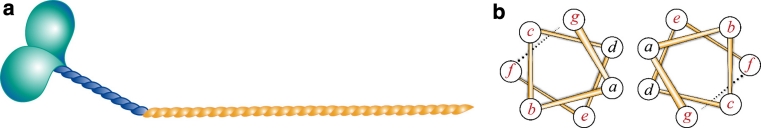
The assembly of MyHC LMM rod domain into coiled-coil α-helices. The heptad repeat motif forms the structural basis for the LMM coiled-coil dimer. The cross-section of the double α-helix coiled-coil with the heptad repeat sequences. While the residues at positions *a* and *d* form the core of the α-helix, the residues at positions *b, c, e, f* and *g* are located at the outer region of the coiled-coil

Mutations in the middle and distal part of the slow/β-cardiac MyHC rod (LMM) region have been associated with several distinct morphological and clinical phenotypes depending on the location of the mutated residue. The assembly of MyHC filaments involves both the proper folding of α-helices into coiled-coils, and the assembly of these coiled-coils into filaments. Defects in any of these steps, caused by mutations, may result in improper filament formation leading to pathological conditions. Coiled-coils are two-stranded protein motifs, where each strand is an *α*-helix with seven-residue repeats (*a*–*b*–*c*–*d*–*e*–*f*–*g*) (Fig. 15). This heptad repeat generally has apolar residues at the *a* and *d* positions. When the two *α*-helical strands wrap around one another, the *a* and *d* positions are internalized and stabilize the structure. Positions *b, c, e, f, g* are exposed on the surface of the protein, where the side chains are available to interact with other proteins, and they may also form intra- and interchain associations that can further stabilize the structure.

Mutations in the LMM region can affect the ability of the protein to form stable and functional thick filaments, based on the amino acid change, the position in the heptad repeat motif and the location in the LMM. Missense mutations associated with myosin storage myopathy (L1793P, R1845W, E1883K and H1901L) are located within or closed to the 29-residue assembly competence domain, in the C-terminus coiled-coil rod region of MyHC, which is known to be critical for the proper assembly of sarcomeric myosin rod filaments [67]. These mutated residues are located either in the outer *b* or *f* position, where the side chains are available to interact with other myosin dimers or other proteins, or in the *d* position that stabilize the structure of the protein. Mutations at outer positions may cause improper filament formation through disturbed interaction with other myosin dimers and thereby perturb thick filament assembly.

A disturbed interaction with other sarcomeric proteins such as titin, myosin-binding proteins (MyBP) C and H, M-protein or myomesin 1, may also be considered. In this context it is of interest to note that one patient with a mutation in the rod region of MyHC I showed cytoplasmic bodies [77], a type of protein aggregates that are otherwise typically associated with A-band titin (*TTN*) mutations [48].

The aberrant accumulations of myosin in the muscle fibers of affected individuals with myosin storage myopathy might be due to improper incorporation of mutated myosin into thick filaments or disassembly of the filaments resulting in accumulation of mutant protein between the myofibrils and beneath the sarcolemma. The observation that some of the accumulated myosin appears to be ubiquitinated indicates that it is marked to be degraded through the proteasomal pathway [17]. The accumulation may be due to insufficient degradation. It is noteworthy that the inclusion bodies are not labeled by p62, which is otherwise common in protein aggregate diseases [86].

Several in vitro studies have been performed to investigate molecular mechanisms involved in the pathogenesis of diseases caused by mutations in the rod region of muscle MyHC. Biochemical and biophysical characterization of the effects of the myosin storage myopathy mutations in the LMM region have suggested adverse effects of the mutations in the ability of the protein to form stable and functional thick filaments [4]. However, analyses of different mutations demonstrated that each mutation has a unique effect on the biochemical and biophysical properties of the LMM [4]. The pathogenic mechanisms of the R1500P and L1706P causing Laing early-onset distal myopathy have been investigated by biochemical assays and in different cellular systems [5, 12]. By co-expression of wild-type and mutant proteins in non-muscle COS-7 cells, in the C2C12 mouse muscle cell line and in neonatal rat ventricular myocytes, dominant adverse effects could be demonstrated including formation of myosin aggregates [12]. Generation of transgenic *C. elegans* indicated that the proline mutations did not affect the thick filament formation or muscle force generation but myosin aggregates were identified in the R1500P mutants [12].

It is still unclear why Laing distal myopathy and myosin storage myopathy are associated with different muscle pathologies. Myosin storage myopathy mutations are located in exons 37–40 in the LMM region of slow/β-cardiac MyHC within or close to the assembly competence domain, which is crucial for proper filament assembly. Consequently, mutations in this region may cause defective integration of dimers into the thick filament leading to accumulation of unassembled MyHC. In contrast, mutations associated with Laing distal myopathy that are situated at distance from the assembly competence domain might cause other effects on the thick filament structure and function leading to different pathology.

## Conclusions

Further work is needed to get insight into the mechanisms governing the cardiac and/or skeletal muscle involvement as well as the pathogenesis leading to protein aggregation in some of the myosin myopathies. For this purpose and for classification of the diseases, which may show clinical overlap, it is essential to perform morphological investigations. Based on correlations between genotype and muscle phenotype in humans, the use of animal models in combination with in vitro studies will allow investigation of the disease mechanisms and clarify the functional impact of mutations affecting different isoforms and different domains of the MyHC.
